# Clinical Decision-Making Case: Intussusception

**DOI:** 10.21980/J8.52171

**Published:** 2025-12-31

**Authors:** Brian Milman, Samuel Parnell

**Affiliations:** * UT Southwestern Medical Center, Department of Emergency Medicine, Dallas, TX

## Abstract

**Audience:**

This clinical decision-making case is intended for emergency medicine residents of all levels.

**Introduction:**

To become board certified in emergency medicine, graduates must pass both a qualifying exam and an oral exam. In 2026, the American Board of Emergency Medicine (ABEM) is transitioning to a Certifying Exam.^1^ Historically, the oral exam included two structured interview cases, now retitled clinical decision-making (CDM) and one pediatric case. Vomiting and abdominal pain are two of the top five reasons pediatric patients present to the emergency department. Being able to take a complete history and exam, regardless of age, and form an appropriate differential diagnosis is a critical skill for emergency physicians. There are many resources available to prepare for standardized single patient encounters, but there are very few resources available for candidates to prepare for the CDM cases. Here we present a CDM case of irritability and vomiting in an 18-month-old for learners to familiarize themselves with the CDM format and to demonstrate management of a pediatric patient.

**Educational Objectives:**

By the end of this mock oral boards session, learners will (1) demonstrate familiarity with the CDM case format and case play, (2) model a problem-based history and physical exam, (3) generate a differential diagnosis for pediatric abdominal pain, and (4) demonstrate the ability to manage intussusception.

**Educational Methods:**

This CDM case is based on the sample script available on the ABEM website. Individual residents were tested by a faculty member virtually via Zoom. After all residents completed the case, a group debrief was held virtually on Zoom.

**Research Methods:**

This case was originally tested with a pilot group of five learners who provided verbal feedback following the case. Adjustments were made to the case based on that feedback. The case was then tested with 36 second- and third-year emergency medicine residents from two residency programs. At the completion of each case, a faculty examiner scored each resident’s performance based on a standardized scoresheet. Residents received one-point for completing each task and the overall score was calculated out of 25 possible points. Residents were surveyed on prior experience with the CDM format and the educational value of this oral boards session. Educational value was evaluated on a 5-point Likert scale with 5 being excellent.

**Results:**

In total, 36 residents completed this mock CDM case. The average score was 20.3/25. All examinees performed palpation on abdominal exam, ordered and provided justification for an abdominal ultrasound, ordered and provided justification for an air contrast enema, and stated the correct diagnosis of intussusception. Nearly all examinees provided an appropriate differential diagnosis for the patient. The most common items that examinees missed included asking about surgical history, asking about blood in the patient’s stool, listening for bowel sounds on exam, and providing at least one vital sign when the inpatient team was called for admission.

Twenty residents responded to the post-case survey (55.6%). When asked if they had previous knowledge of the CDM format, only 30% of respondents were aware of this case format, and only 10% of respondents had previously participated in a CDM practice case. The learners rated the educational value of this case a 4.7/5 with 95% “agreeing” or “strongly agreeing” that the case was helpful in preparing for their oral board exam and 90% feeling like the educational value was “very good” or “excellent.”

**Discussion:**

We present a CDM case of intussusception that allows the resident to become familiar with this type of case while demonstrating their ability to obtain a history from, examine, and treat a pediatric patient. Through this case, residents are asked specific questions about the thought process behind the history and physical exam they perform. They are also required to provide a differential diagnosis, treatment plan, and disposition for the patient. Through this clinical decision-making process, our residents felt that the case was of high educational value and was helpful in preparing for the certifying exam.

**Topics:**

Structured interview, clinical decision-making, pediatric abdominal pain, intussusception, certifying exam.

## USER GUIDE

**Table:** 

List of Resources:	
Abstract	148
User Guide	150
For Examiner Only	152
Certifying Exam Assessment	158
Stimulus	160
Debriefing and Evaluation Pearls	164

**Learner Audience:**

Interns, Junior Residents, Senior Residents

**Time Required for Implementation:**

Case: 15 minutes

Debriefing: 10 minutes

**Recommended number of learners per instructor:**

1

**Topics:**

Structured interview, clinical decision-making, pediatric abdominal pain, intussusception, certifying exam.

**Objectives:**

By the end of this CDM case, learners will be able to:

1. Demonstrate familiarity with the CDM format and case play.
2. Model a problem-based history and physical exam.
3. Generate a differential diagnosis for pediatric abdominal pain.
4. Demonstrate the ability to manage intussusception.

### Linked objectives, methods and results

The primary objective of this case is to introduce learners to the CDM case format. The best way to ensure familiarity with the format is to have learners work through a mock CDM case. Many emergency medicine residencies incorporate oral boards style cases into their curriculum through Foundations of Emergency Medicine or mock oral boards sessions. However, only 30% of the upper-level residents we surveyed were aware of the CDM case format. Using constructivism, we facilitate residents expanding their current knowledge of the oral boards case format to one that includes the CDM cases. Additionally, the CDM format utilizes a behavioral learning framework by providing positive and negative feedback to the learner throughout the case. The learner is first asked to list all the historical information they would like to obtain. The learner is then provided with a list of historical information. If their history-taking was complete, they receive positive reinforcement by getting the information they requested. If their list was incomplete, they are immediately reminded of historical information that they forgot. This behavioral learning process continues through the physical exam and diagnostic studies. With bedside teaching, it is difficult for a learner to explain why they are asking certain questions or performing certain elements of the exam in real time. However, the CDM format allows the facilitator to understand the learner’s decision-making process.

### Recommended pre-reading for instructor

Instructors should view the sample clinical decision-making video prior to the case to ensure that they are familiar with the CDM case format. These videos and other helpful certifying exam preparation resources can be found on the ABEM website. Accessed: November 17, 2025. Certifying Exam Content: https://www.abem.org/get-certified/certifying-exam/certifying-exam-content/

### Results and tips for successful implementation

During the 2022–2023 academic year, this case was piloted with five third-year emergency medicine residents, individually with a single faculty member facilitator. Each resident was asked for verbal feedback following the case. In the original case, an ultrasound report was given as part of Stimulus 2. Following discussion with the pilot group, the case was modified to include the ultrasound images rather than the report. The modified case was then tested on April 11, 2024, with 36 residents from two emergency medicine residency programs. Of these, 33 were third-year residents and three were second-year residents. This case has not been tested with any interns. Each resident was paired 1:1 with an emergency medicine faculty facilitator in a Zoom breakout room. The resident had 15 minutes to complete the case. At the completion of the case, the faculty member scored the resident using the scoresheet below and provided written feedback. The average score was 20.3/25. Residents were surveyed to determine their experience with the CDM case format and the educational value of the case. Twenty residents completed the post-case survey (response rate of 55.6%). Of the respondents, only 30% were aware of the CDM case format, and only 10% had previously participated in a CDM practice case. On a Likert scale from 1 (Poor) to 5 (Excellent), participants rated the educational value as 4.7. Following completion of the case with all learners, we convened as a group and debriefed the case to reinforce the learning objectives for all learners.

## Figures and Tables

**Table:** 

	Mild 3–5%	Moderate 6–10%	Severe >10%
Mental Status	Normal	Listless, irritable	Lethargy, altered mental state
Heart Rate	Normal	Increased	Increased
Pulses	Normal	Normal to decreased	Decreased to thready
Capillary Refill	Normal	Prolonged	Prolonged
Blood Pressure	Normal	Normal	Normal to decreased
Respirations	Normal	Tachypnea	Tachypnea
Eyes	Normal	Slightly sunken, decreased tears	Sunken, cries without tears
Fontanelle	Normal	Sunken	Sunken
Urine Output	Normal to decreased	Decreased	Oliguric or anuric

**Table:** 

I. History		Yes	No
**1**	Surgical history		
**2**	Time course		
**3**	Blood in stool		
**4**	Provides rationale		
**II. Physical Examination**			
**5**	Auscultate abdomen		
**6**	Palpate abdomen		
**7**	Visual inspection of scrotum		
**8**	Provides rationale		
**III. Differential Diagnosis**			
**9**	Diagnosis 1 - *Examples of diagnoses listed in differential diagnosis scoring guidelines above*		
**10**	Diagnosis 2 - *Examples of diagnoses listed in differential diagnosis scoring guidelines above*		
**11**	Diagnosis 3 - *Examples of diagnoses listed in differential diagnosis scoring guidelines above*		
**IV. Diagnostic Studies**			
**12**	BMP or CMP		
**13**	Ultrasound Abdomen		
**14**	Rationale for ultrasound		
**15**	Rationale for not ordering CT		
**V. Treatment and Other Actions**			
**16**	IV crystalloid		
**17**	Air contrast enema		
**18**	Rationale for fluids		
**19**	Rationale for enema		
**VI. Broad Principles**			
**20**	Assessment of pediatric dehydration		
**VII. Final Diagnosis**			
**21**	Intussusception		
**VIII. Disposition**			
**22**	Admit to floor		
**23**	Rational for admission/discharge		
**IX. Transitions of Care**			
**24**	Report the diagnosis		
**25**	Provide at least one vital sign or report that vital signs were normal		
**26**	Provide clear information on treatment/interventions		

**Table:** 

**Candidate Task Sheet**	
**#1**	**Emergency Department Admitting Form**
**#2**	**Diagnostic Studies: Pertinent Positives/Negatives**

**Table:** Clinical Decision-Making Candidate Task Sheet

**CASE PARAMETERS**
This is a 15-minute case You will interact with two examiners. This is an interview style without role playing; you should simply reply to the questions asked. You may be interrupted to move you through the case; this is not a reflection of your performance.
**PATIENT INFORMATION**
An 18-month-old male is brought in by his mother for irritability and vomiting. Symptoms have been intermittent for the past 8 hours. The patient appears uncomfortable. Mother describes his stool as slimy. The patient has had decreased oral intake and has no previous medical problems.
**VITAL SIGNS**
BP: 92/50 mmHg, P: 130/min, R: 30/min, T: 37.0°C, 02 Sat: 98%
**TASK STATEMENT**
Your tasks are as follows: List pertinent elements of a focused history and physical exam Develop an appropriate differential and/or provisional diagnosis Select and interpret appropriate studies Articulate appropriate patient management including discharge instructions

**STIMULUS 1:** Emergency Department Admitting Form

**Patient Information**	
**Age**	18 months
**Gender**	Male
**Method of Arrival**	Private vehicle
**Chief Complaint**	Irritability and vomiting
**Person Providing History**	Patient’s mother
**General Appearance/History of Present Illness**	Intermittent fussiness and vomiting for the past eight hours Appears uncomfortable Mother describes stool as “slimy” Decreased oral intake No previous medical problems
**Vital Signs on ED Arrival**	Temp: 37.0° C (98.6° F), rectal BP: 92/50 mmHg P: 130/min RR: 30/min O2 sat: 98%

**STIMULUS 2:** Diagnostic Studies: Pertinent Positives/Negatives

**CBC**	WBC	10,000/mm^3^
	Hemoglobin	14.2 g/dL

**BMP**	Na	141 mEq/L
	K	4.3 mEq/L
	Cl	110 mEq/L
	C02	20 mEq/L
	BUN	18 mg/dL
	Creatinine	0.7 mg/dL

**UA**	Ketones	neg
	Leuk. Est.	neg
	Nitrite	neg
	WBC	0–1/HPF

**Toxicology**	Negative	

**US Abdomen4**	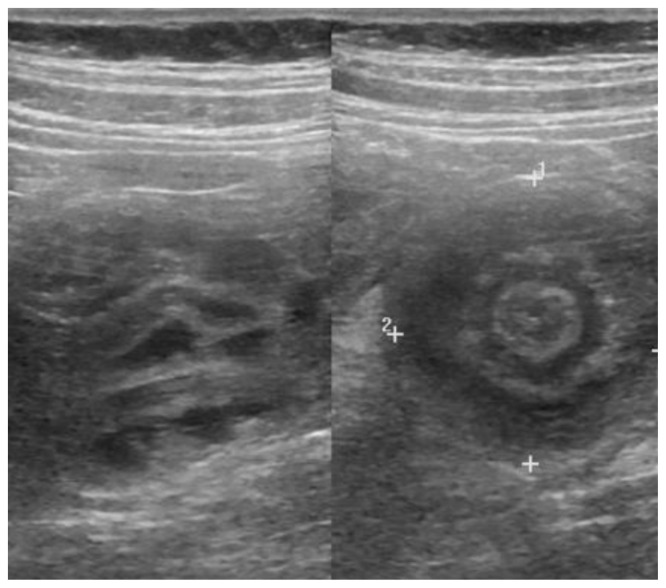	

## FOR EXAMINER ONLY

### Clinical Decision-Making Case: Intussusception Summary

**Diagnosis:** Intussusception

**Case Summary:** This is a case of an 18-month-old male presenting with eight hours of irritability and vomiting. The patient was born at term and has no previous medical problems or surgeries. He has been having intermittent episodes of crying and vomiting. The episodes last 20 minutes, then resolve, and patient is comfortable until episodes recur. He has had decreased oral intake and some red tinged stool that the mother describes as “slimy.” On exam, the patient is crying, appears dry, grimaces with abdominal palpation, and has diminished bowel sounds.

**Synopsis of Physical Exam:**

T 37.0°C (rectal), BP 92/50 mmHg, HR 130/min, RR 30/min, Pulse Ox 98% on room air.

Crying on exam

Dry mucous membranes

Grimaces with abdominal palpation

Bowel sounds are diminished

Bloody stool in diaper

Normal testicular exam

Normal capillary refill

### Clinical Decision-Making Case: Intussusception Examiner Script

**Case Introduction:**

“Hello Doctor, this is a clinical decision-making case. There is no role playing. In response to the questions I will ask, please give me a LIST of information you would gather to come to a final diagnosis. At times, I may interrupt you to move you through the case; this is not a reflection of your performance. You will have 15 minutes to complete the case. Before we begin, do you have any questions?”

“The patient we will be discussing is an 18-month-old male with irritability and vomiting for the past eight hours.”

Provide Learner Stimulus #1

### HISTORY

Prompt 1:

“Here is the initial information regarding this patient. After you have read it, please give me a list of the additional historical information you would obtain.”

Prompt 2:

“You indicated you would ask the patient about X. Why is this important to you?”

Examples: Blood in stool, time course of symptoms, birth history
*This response will depend on the learner’s questions and will ultimately be chosen by the interviewer.*

Scoring Guidelines:

4. Rationale: Blood in the stool indicates GI etiology and can be seen with intussusception, Meckel’s diverticulum, trauma, volvulus.

General Guidelines:

- If candidate begins managing the case like a standard case, examiner states, “Remember Doctor, there is no role playing in this case. Please list the additional information you want to obtain.”
- If candidate does not offer a complete list of historical information, examiner should pause long enough to allow them to list additional items, before asking “why” questions.
- If candidate mentions “past medical history,” or “social history” examiner clarifies by asking, “What specifically do you want to know about PMH/social history?”

### PHYSICAL EXAMINATION

“You are provided with the following additional historical information:”

- Born at term
- Episodes of crying and vomiting last 20 minutes. The patient then seems comfortable for an hour or so but then another episode occurs.
- Red-tinged stool with one diaper change
- No previous surgeries
- No recent travel
- Not in daycare

Prompt 3:

“Based on what you now know, please describe the physical exam that you would perform.”

Prompt 4:

“Doctor, you examined X during the physical exam. Please explain how that would help you.”

Examples: Abdominal palpation, testicular exam, oropharyngeal exam
*This response will depend on the learners’ questions and will ultimately be chosen by the interviewer.*

Scoring Guidelines:

8. Rationale: Assess location of pain, presence of masses, and peritoneal signs

### DIFFERENTIAL DIAGNOSIS

“You are provided with the following physical exam findings:”

- Crying in mother’s arms
- Slightly dry mucous membranes
- Grimaces with abdominal palpation
- Bowel sounds are diminished
- Bloody stool in diaper
- Normal testicular exam
- Normal capillary refill

Prompt 5:

“Based on what you now know, what are the top three items on your differential diagnosis based on the most likely conditions?” (If more than three conditions are mentioned, say, “OK thank you. Please give me your top three, and only three, most likely diagnoses.”)

Appropriate differential diagnoses include:

- Intussusception
- Volvulus
- Bacterial colitis
- Meckel diverticulum
- Appendicitis
- Trauma
- Testicular torsion

Scoring Guidelines:

Rationale: Mentioning any diagnoses above. Other pathologies that present at 18 months of age and cause intermittent abdominal pain, vomiting, or blood in stool may also be counted.

### DIAGNOSTIC STUDIES

Prompt 6:

“Based on what you know and your working differential diagnosis, what, if any, diagnostic studies would you order?”

Prompt 7:

“Doctor, you ordered X. Why X?”

“Doctor, you did not order a CT abdomen/pelvis. Why wouldn’t you want a CT scan in this patient?”

Scoring Guidelines:

14. Rationale: Ultrasound is diagnostic with high sensitivity and specificity.

15. Rationale: Radiation exposure - ultrasound is available and has similar diagnostic accuracy.

*After the candidate responds,*
Provide Learner Stimulus #2

### TREATMENT AND OTHER ACTIONS

Prompt 8:

“Based on what you now know, what treatments, if any, would you order and/or what actions, if any, would you perform?”

Prompt 9:

“Doctor, you ordered X. Why X?”

### ASK THIS PROMPT TWICE ABOUT TWO SEPARATE TOPICS

Example: fluids, air contrast enema

Scoring Guidelines:

18. Rationale: Evidence of volume contraction given physical exam and BMP.

19. Rationale: Definitive treatment

### BROAD PRINCIPLES

Prompt 10:

“Doctor, how do you determine the degree of dehydration in a pediatric patient?”

Scoring Guidelines:

20. Rationale: The examinee needs to mention four or more of the below clinical parameters and needs to mention how to determine the difference between mild and moderate or severe for at least one clinical parameter.

### FINAL DIAGNOSIS

Prompt 11:

“Based on everything you know about this case, what is your final diagnosis?” Intussusception

Scoring Guidelines:

21. Rationale: Verbalizing intussusception meets the critical action. If the candidate mentions “bowel obstruction,” the examiner asks, “can you be more specific about the diagnosis?”

### DISPOSITION

Prompt 12:

“Based on what you know, what should be the disposition of this patient?”

Prompt 13:

“Why would you [admit/discharge] this patient?”

Scoring Guidelines:

22. Rationale: Admit for air contrast enema, hydration, observation.

### TRANSITION OF CARE

Prompt 14:

[If admit] “The admitting team is on the phone and would like sign-out on this patient.”

[If discharge] “Please provide verbal discharge instructions to the patient’s parent.”

Scoring Guidelines:

23–25. Rationale: Appropriate handoff to the admitting team or discharge instructions. Must include diagnosis and some mention of vital signs if admitted. Must include diagnosis and return precautions if discharged.

*Thank you, Doctor. That concludes this case.*

*Please tear up your notes*

## CERTIFYING EXAM ASSESSMENT

*Clinical Decision-Making Case: Intussusception*

Learner: _________________________________________

**Summative and formative comments:**

## DEBRIEFING AND EVALUATION PEARLS

### Clinical Decision-Making Case: Intussusception

#### Case Overview

This is a case of pediatric vomiting and irritability. Through information provided by the patient’s mother, a thorough physical examination, and ultrasonography, the diagnosis of intussusception is made. Radiology is consulted for reduction with air enema.

#### The Pediatric Patient

Data gathering is critical for patients who cannot articulate for themselves. Rapid assessment using appearance, work of breathing, and circulation to skin helps determine if this is a patient that requires immediate resuscitation or if there is time to move into a thorough history and exam. Use a reference or your institution’s electronic medical record (EMR) to determine if vital signs are appropriate for age.

#### Pediatric Abdominal Complaints

The etiology of abdominal complaints in the pediatric population varies by age and presentation. Irritability, poor feeding, vomiting, absence of stool, bloody stool, fever may all indicate an underlying abdominal pathology.^6^ Red flags include bilious emesis, lethargy, severe pain or irritability, peritoneal signs, abdominal distension, or sepsis.^6^

#### Initial Differential Diagnosis

The differential diagnosis of irritability, vomiting, and abdominal pain in children 3-months-old to 3-years-old includes intussusception, testicular torsion, trauma, volvulus, appendicitis, bacterial colitis, toxic megacolon, vaso-occlusive crisis, gastroenteritis, constipation, urinary tract infection, Henoch-Schonlein purpura. The episodic time course of the patient’s pain should move intussusception to the top of the differential.

#### Management

Ultrasound is the diagnostic test of choice and is 98–100% sensitive.^3^ In patients with signs of perforation, shock, or peritonitis, emergent surgical consultation is necessary.^3^ When these conditions are absent, reduction with air, saline, or contrast enema should be performed by a trained radiologist. Success of non-surgical reduction is >90%.^3^

#### Admission and Consultation

Indications for admission include need for surgical management, difficult or failed enema reduction, significant dehydration. 3 In the absence of these factors, discharge may be considered after a period of ED observation following successful enema reduction and tolerance of oral intake. However, in most emergency departments that do not have pathways for expedited care of patients with intussusception, admission is required.
